# Reduction in left atrial and pulmonary vein dimensions after ablation therapy is mediated by scar

**DOI:** 10.1016/j.ijcha.2021.100894

**Published:** 2021-10-23

**Authors:** Lisa A. Gottlieb, Nora Al Jefairi, Dounia El Hamrani, Jérôme Naulin, Jérôme Lamy, Nadjia Kachenoura, Marion Constantin, Bruno Quesson, Hubert Cochet, Ruben Coronel, Lukas R.C. Dekker

**Affiliations:** a Electrophysiology and Heart Modeling Institute, University of Bordeaux, Pessac, France; b Department of Experimental Cardiology, AUMC, Academic Medical Center, Amsterdam, the Netherlands; c Department of Cardiac Pacing and Electrophysiology, University Hospital, Bordeaux, Pessac, France; d Department of Radiology and Biomedical Imaging, Yale University, New Haven, USA; e Sorbonne Université, CNRS, INSERM, Laboratoire d’Imagerie Biomédicale, LIB, Paris, France; f Department of Electrical Engineering, University of Technology, Eindhoven, the Netherlands; g Cardiology Department, Catharina Hospital, Eindhoven, the Netherlands

**Keywords:** Atrial fibrillation, Ablation scar, Pulmonary vein isolation, Cardiac magnetic resonance, Atrial contractility

## Abstract

**Background:**

Ablative pulmonary vein isolation (PVI) decreases pulmonary vein (PV) and left atrial (LA) dimensions in atrial fibrillation (AF) patients. These changes are attributed to reverse structural remodeling following sinus rhythm restoration but evidence is lacking. We hypothesized that the downsizing is directly caused by the ablative energy and subsequent scar formation.

**Methods:**

We studied cardiac magnetic resonance imaging in 21 paroxysmal AF patients before and 3 months after successful PVI and in healthy sheep (n = 12) before and after PVI of the right PV only.

**Results:**

PVI decreased the PV diameter in patients and sheep by 11.0(10.3) and 9.2(11.0)%, (p < 0.001 and p = 0.020), respectively. The control left PV in sheep were unchanged. A linear correlation existed between the extent of PV scar and PVI-induced decrease in PV diameter in patients.

After PVI, the LA volume decreased (103(38) *vs.* 92(31)ml, pre- *vs.* post-ablation, respectively, p = 0.006), while the right atrial (RA) volume was unchanged in patients. A decrease in active emptying fraction after ablation (26.5(10.7) *vs.* 21.8(10.6)%, pre- *vs*. post-ablation, p = 0.031) was associated with reduced contractility of the PV walls (p = 0.004). The contractility of the LA walls was unaltered (p = 0.749).

**Conclusion:**

The ablation-induced PV diameter reduction was similar in patients with AF and healthy sheep without AF and was associated with PV scar extent. The volume only decreased in LA and not RA after PVI, and wall contractility decreased only in ablated sites. Therefore, the PVI-induced atrial downsizing is caused by the ablative energy and subsequent scar formation.

## Graphical abstract

The reduction in LA and PV dimensions after ablation. Clinically successful PVI ablation decreased the PV and LA dimensions in AF patients (left panel). We make observations (right panel) that are compatible with the direct effect of ablation energy and subsequent scar formation causing the atrial downsizing.

**Figure:**
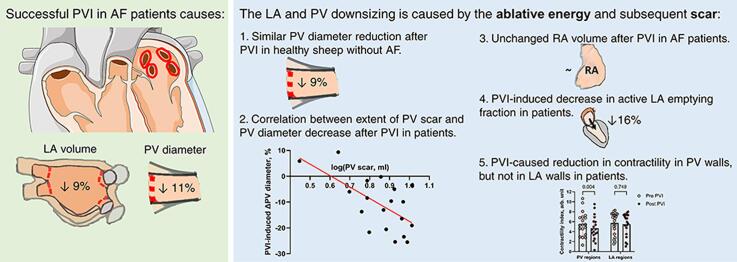

## 1 Introduction

Atrial fibrillation (AF) initiates a structural remodeling of the atrial myocardium involving loss of contractile fibers and an increase in interstitial collagen accumulation (fibrosis) [1], [2]. Often, AF patients have enlarged atria and a reduced atrial contractile function [3]. Ablative pulmonary vein isolation (PVI) is a therapeutic option for AF when pharmacological therapy fails and is recommended in drug-resistant patients with paroxysmal AF (episodes of AF lasting <1 week) [4]. On long-term follow-up, PVI cures 59% of paroxysmal AF patients. [5]

Successful PVI ablation causes a decrease in PV and left atrial (LA) dimensions within months after the ablation procedure. [6], [7], [8] This reduction in PV and LA dimensions is attributed to a decrease in AF burden and thereby reverse structural remodeling in the atria. [9], [10], [11], [12] Reverse remodeling is a return towards the pre-disease state and involves the decrease of structural (hypertrophy, fibrosis, and atrial dilatation) and functional (decreased contractility) abnormalities. [13] However, there is no evidence underlying the association between the PVI-induced reduction in atrial dimensions and reverse remodeling on the one hand, and the subsequent scar formation on the other.

We hypothesized that the effect of ablative energy and subsequent scar formation explain the reduction in PV and LA dimensions. With cardiac magnetic resonance (CMR), we therefore studied PVI-induced changes in 1) PV dimensions, atrial volumes and LA contractility in AF patients with clinically successful ablation and 2) PV dimensions in healthy sheep without AF. We anticipated that reverse structural remodeling initiated by normalization of the cardiac rhythm 1) only occurs in the setting of AF remodeling, 2) similarly takes place in the LA and right atrium (RA), 3) is characterized by an increase in contractile fibers and thereby contractility, and 4) exert an effect on LA dimensions in all cardiac phases. We tested this in patients with paroxysmal AF that were successfully treated with PVI ablation and in sheep without AF, and provide evidence that the changes in LA and PV diameter are the result of the ablative energy delivery and subsequent scar formation and not of reverse remodeling induced by decreased AF-burden.

## 2 Methods

### 2.1 Patient selection

Fifty-one paroxysmal AF patients referred for a first PVI ablation included in a previously published study [14] were retrospectively analyzed. The study was approved by the Institutional Ethics Committee (Comité de Protection des Personnes Sud‐Ouest et Outre Mer III, approval reference 2012‐A01494‐39). Informed consent was obtained from all patients. All patients were re-catheterized 3 months after the ablation procedure to test for recurrence of electrical PV-LA conduction. We included only patients with a clinically successful PVI defined as presence of sinus rhythm during the post-ablation CMR and re-catheterization both scheduled 3 months after the ablation procedure. Additional exclusion criteria were comorbidity of valvular disease, heart failure, administration of ablative radiofrequency (RF) outside the PV, AF during pre-ablation CMR. Twenty-one paroxysmal AF patients were included of whom 7 were female (33%). Their age was 62(9) years and body mass index was 26.6(3.5) kg/m^2^. AF diagnosis had been made 60[60] months previously.

### 2.2 Patient CMR acquisition

CMR studies were conducted on a 1.5 Tesla system (MAGNETOM AVANTO, Siemens, Erlangen, Germany) equipped with a 32-channel body coil and a 18-channel cardiac coil. Cine imaging was performed by ECG-gated steady-state free precession pulse sequence during breath-holding in order to acquire a stack of 4-chamber images covering the entire heart (slice thickness 6 mm without interslice gap) as well as a 2-chamber slice (slice thickness 6 mm; temporal resolution: 15 ms).

A late gadolinium enhancement (LGE) CMR acquisition was begun 17 min after intravenous injection of 0.2 mmol/kg gadoterate meglumine (Guerbet, Aulnay-sous-bois, France) and consisted of a *trans*-axial three-dimensional orientation using an ECG-gated and respiration-navigated gradient-echo pulse sequence.

The following imaging parameters were used: in-plane resolution = 1.25 mm× 1.25 mm; flip angle = 22°; echo time = 2.4 ms; repetition time = 6.1 ms; generalized auto-calibrating partial parallel acquisition (GRAPPA), acceleration factor of 2 with 42 reference lines. The pre-ablation CMR was performed within 3 days before the ablation procedure, while the post-ablation CMR was performed 91 ± 29 days after the pre-ablation CMR.

### 2.3 Patient ablation procedure

The ablation procedure has previously been described in depth. [14] Shortly, patients were randomized to complete PVI ablation with either a conventional point catheter (NAVISTAR THERMOCOOL, Biosense Webster, Irvine, CA, United States; n = 11/21) or a circular catheter (nMARQ, Biosense Webster; n = 10/21). Endocardial LA access was achieved by *trans*-septal puncture and followed by heparin administration (0.5–0.8 mg/kg). RF energy was delivered for 60 s per application in a unipolar mode with a temperature limitation of 45 °C and simultaneous irrigation with 0.9% saline (60 mL/min). All patients had successful immediate procedural PVI confirmed by activation mapping that persisted after adenosine administration. We consider that the variations in lesion localization within catheter type differ equally when comparing both types.

### 2.4 Sheep CMR acquisition

The sheep study was carried out in accordance with the EU Directive 2010/63/EU for protection of animals used for scientific purposes and approved by the local ethical authorities at University of Bordeaux, France (approval number 7995).

CMR acquisition was performed *in vivo* in healthy female sheep (n = 12, 2–3 years old, 54(4) kg; sheep strain: Charmoise) before and 3–4 months after RF ablation of the right pulmonary vein (RPV). The sheep were anesthetized (pre-medication: 20 mg/kg ketamine + 0.1 mg/kg acepromazine, induction: 1 mg/kg propofol, maintenance: 2% isoflurane) before placed on their back on the scanner table. The CMR studies were conducted on a 1.5 Tesla system (MAGNETOM Aera, Siemens, Erlangen, Germany) equipped with a 32-channel body coil and an 18-channel cardiac coil. Cine imaging was performed by ECG-gated steady-state free precession pulse sequence during forced breath-hold in order to acquire a stack of *trans*-axial images. Slice thickness was 4 mm (n = 7) or 6 mm (n = 5) and did not differ between pre- and post-ablation CMR in the same animal.

The following acquisition parameters were used: in-plane resolution = 1.3 mm × 1.3 mm; flip angle = 58°; bandwidth = 992 Hz/pixel; echo time = 1.34 ms; repetition time = 21.98 ms; GRAPPA; acceleration factor of 3 with 75% partial Fourier acquisition. The pre-ablation CMR was performed within 1 week before ablation, while the post-ablation CMR was performed 120 ± 11 days after the pre-ablation CMR.

### 2.5 Sheep ablation procedure

The sheep were catheterized via the femoral veins under general anesthesia and sterile conditions. LA access was achieved by *trans*-septal puncture using a steerable sheath. A circular ablation catheter (PVAC Gold, Medtronic, Minneapolis, MN, United States) was placed in the RPV ostium under fluoroscopy guidance, and RF energy was administered 2 × 60 s with bipolar:unipolar 2:1 phasing and a temperature limitation of 55 °C. The ablation reference electrode was placed on the lower back of the sheep. The animals recovered under surveillance for 1 week before returning to a hosting farm.

### 2.6 Image analysis

**Fig. 1:**
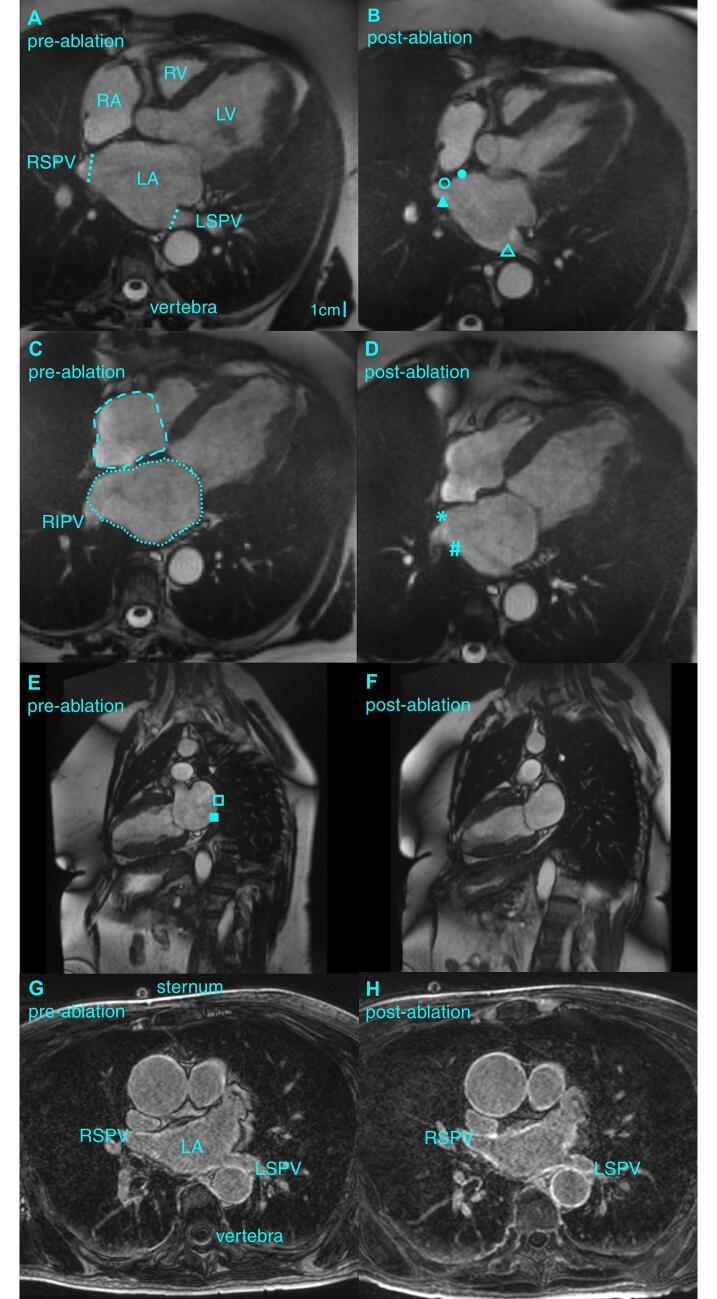
CMR images in AF patients. Four-chamber slice with RSPV and LSPV at the moment of maximum LA dilatation in patients before (A) and after (B) PVI ablation. Dotted lines: PV diameters. Closed circles: LA septal wall. Open circles: RSPV anterior wall. Closed triangle: RSPV posterior wall. Open triangle: LSPV posterior wall. Four-chamber slice with RIPV before (C) and after (D) ablation. Dotted lines: RA and LA surface areas for volume estimation. Asterisks: RIPV anterior wall. Hashtag: RIPV posterior wall. Two-chamber slice before (E) and after (F) ablation. Open square: LA posterior wall. Closed square: LA inferior wall. LGE CMR before ablation (G) and after ablation (H). The scar formation in the PV walls after ablation is noted.

**Supplementary Fig. 1:**
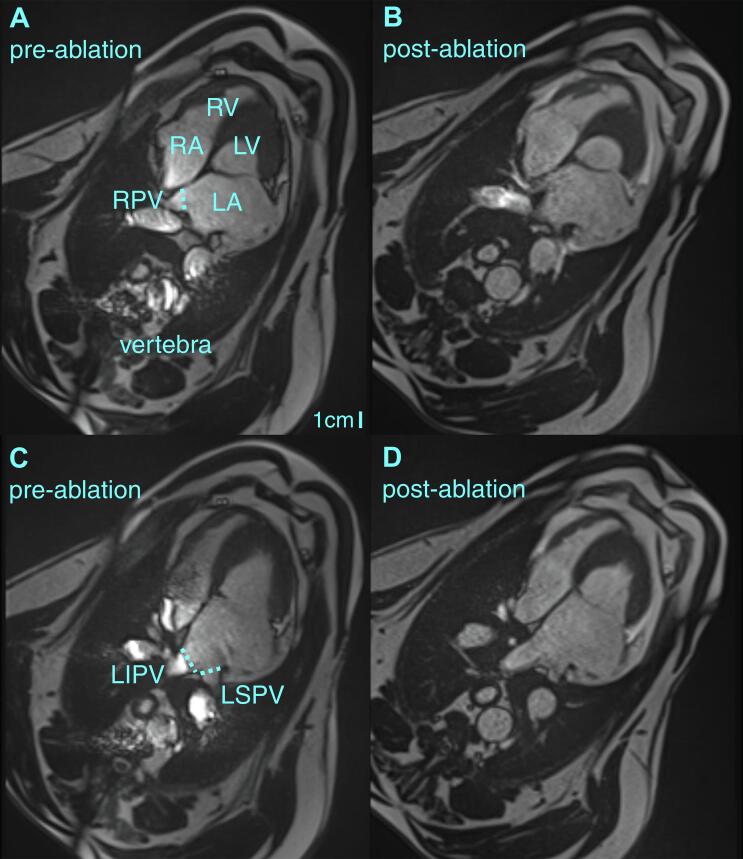
Cardiac magnetic resonance images in healthy sheep. *Trans*-axial slice with RPV at the moment of maximum LA dilatation in sheep before (A) and after (B) localized RPV ablation. Slice with LIPV and LSPV before (C) and after (D) ablation. Dotted lines: PV diameters.

Only pre- and post-ablation images containing the same anatomical landmarks (the sternum, the vertebrae, and the aorta) in similar stack slices and being without artefacts in the LA and PV regions were included in the analysis that was performed in the software Syngo.via (Siemens, Erlangen, Germany). The PV diameter was measured at the ostium at the moment of maximum LA dilatation (just before opening of the mitral valve) in the patients (Fig. 1A) and sheep (supplementary Fig. 1) if the PV remained temporally in plane in both pre- and post-ablation images.

In the patients, we analyzed the LA volume at the moment of maximum LA dilatation, and immediately before (pre-contraction) and after (minimum LA volume) atrial contraction by tracing the endocardial LA contours on each slice of the 4-chamber stack while excluding the PV and LA appendage (LAA) Fig. 1C). The volume was calculated as the sum of each LA area multiplied by the slice thickness. The maximum RA volume was similarly measured while excluding the caval veins from the RA areas (Fig. 1C).

We defined the total LA ejection fraction as (maximum LA volume – minimum LA volume)/maximum LA volume × 100%, the LA expansion index as (maximum LA volume – minimum LA volume)/minimum LA volume × 100%, the LA conduit fraction as (maximum LA volume – pre-contraction LA volume)/maximum LA volume  × 100%, and the LA active emptying fraction as (pre-contraction LA volume – minimum LA volume)/pre-contraction LA volume × 100%.

We analyzed local LA strain and motion fraction throughout the cardiac cycle with a feature-tracking algorithm previously described. [15], [16] LA endocardial markers (>30) were positioned on a single phase of the cardiac cycle, and longitudinal strain (tangential deformation to the considered LA wall) and radial motion fraction (towards the LA center of mass) of defined regions were computed. We identified the anterior and posterior wall of the right superior PV (RSPV) and right inferior PV (RIPV) as well as the posterior wall of the left superior PV (LSPV; Fig. 1B). The tracking of the slightly kinked anterior LSPV wall towards the LAA was less precise and therefore excluded from the analysis. Also, 3 LA regions outside the PV were defined: a septal LA wall in the 4-chamber stack (in the slice with the RSPV; Fig. 1B) and a posterior and an inferior LA wall in the 2-chamber slice (Fig. 1E). The PV ostia and LAA were excluded from these regions. The temporal beginning of the strain/motion fraction curve was the moment immediately after atrial contraction, and we considered the maximum magnitude of the last 20% of the curve as an indicative of local contractility. A contractility index of the PV regions and of the LA regions was calculated in each patient as the sum of the logarithmically transformed values of local contractility (2 values per wall region: 1 value each from the strain and the motion fraction curve. The PV contractility index was calculated based on the 5 PV regions; the LA contractility index on the 3 LA regions).

Scar burden was quantified on the LGE CMR images (Fig. 1G) using the MUSIC software (IHU Liryc, Bordeaux, France) as previously described. [14] Shortly, the LA wall was manually traced, and LGE was segmented using histogram analysis, applying the full width at half maximum method. Thresholding of myocardial voxel intensity (50–70% of maximum signal intensity) was performed, and scar in the PV and LA was quantified as the difference between pre- and post-ablation measurements and expressed in ml.

### 2.7 Histology

Three sheep hearts were explanted after post-ablation CMR acquisition in accordance with the ethically approved euthanasic methods. The RPV and left PV (LPV) were dissected and fixated in paraformaldehyde (4%) at 5 °C for 2 weeks before cut in the longitudinal direction thus including the atrial-PV junction and the distal PV. Specimen were dehydrated automatically (Leica HistoCore Pearl processor, Wetzlar, Germany) before being embedded in paraffin and sectioned with a microtome (6 μm slice thickness). A Masson’s trichrome staining was applied to the slices to visualize cardiomyocytes (red), nuclei (black), and collagen (green) before digitally scanned with an objective × 20.

### 2.8 Statistical analysis

Data are expressed as mean (standard deviation) or median [interquartile range] depending on normality tested with a Shapiro Wilk’s test. Pre- and post-ablation CMR data were tested with a two-tailed paired Student’s *t*-test or a Wilcoxon signed rank test, as appropriate. P-values < 0.05 were considered as statistically significant. The 95% confidence intervals (CI_95%_) are included for parametric testing.

A mean of ablation-induced change in PV diameters (post-ablation diameter – pre-ablation diameter / pre-ablation diameter  × 100%) was calculated in each patient and was tested against a theoretical mean of 0 with a two-tailed one-sample Student’s *t*-test. The change in diameter after ablation of the ablated RPV and a mean change in diameter of the non-ablated LPV were calculated in each sheep before similar testing and Bonferroni correction (0.05/2). A two-tailed unpaired Student’s *t*-test was used to test for differences in ablated PV between patients and sheep.

A linear regression model was fitted to the mean PV diameter change in patients as a function of the logarithmically transformed extent of PV ablation scar. A mixed effects linear model was applied to the PV and LA contractility indexes.

## 3 Results

Not all PV were accessible for analysis. The left inferior PV (LIPV) in the AF patients was rarely visible and comparable between the pre- and post-ablation datasets (n = 4) and was discarded from the analysis. In patients, 14 RIPV, 18 RSPV, and 16 LSPV were analyzed. None had left or right single ostium PV or unilateral triple PV. In sheep, 11 RPV, 10 LSPV, and 11 LIPV were measured (a RIPV is absent in sheep).

### 3.1 Ablation-caused decrease in PV diameter

**Fig. 2:**
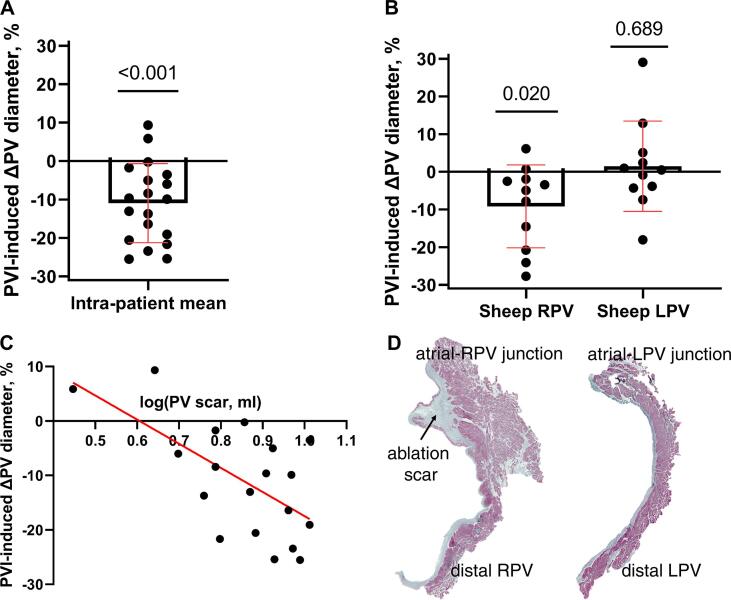
Ablation-induced changes in PV diameter and scar. A: The PV diameter decreased after PVI. A mean of ablation-induced PV diameter change was calculated in each patient and hypothesis-tested against a theoretical mean of 0 (one sample Student’s *t*-test). B: The diameter of the ablated RPV decreased after ablation in sheep, while the diameter of the control LPV (intra-sheep mean) was unchanged. C: There existed a negative linear correlation between PV diameter change and extent of PV scar (logarithmically transformed). D: Collagenous ablation scar occurred in the RPV (left panel), but not in the LPV of sheep (right panel).

Patients: PVI ablation caused a decrease in PV diameter in the AF patients (18.9(4.2) *vs.* 16.5(3.8) mm, pre- *vs.* post-ablation, respectively; PVI-induced change: −11.0 (10.3) %, CI_95%_=[-15.9–6.0], p < 0.001, hypothesis test against theoretical mean equaled 0; Fig. 2A)

Sheep: The PV diameter of the ablated RPV in sheep also decreased after ablation (13.8(2.3) *vs.* 12.3(1.6)mm, pre- *vs.* post-ablation, respectively; PVI-induced change: −9.2(11.0)%, CI_95%_=[-16.6–1.8], p = 0.020; Fig. 2B), whereas the control left PV remained unchanged (15.6(2.3) *vs.* 15.5(2.2)mm, pre- *vs.* post-ablation, respectively; PVI-induced change: 1.5(12.0)%, CI_95%_=[-6.6 9.5], p = 0.689; Fig. 2B).

The percentage decrease in ablated PV diameter was similar (-11.0(10.3) and −9.2(11.0)% in patients and sheep, respectively, CI_95%_=[-6.4 10.0], p = 0.660).

### 3.2 PV scar

Patients: Three months after PVI, ablation scar was observed in the PV regions in all patients (7.4 (2.1)ml; Fig. 1H). LA scar was observed in 1 patient (1.5 mL). A negative linear relation existed between ablation-induced PV scar and PV diameter change (R^2^ = 0.388, H_0_ slope equal to 0: p = 0.004, slope = -44.3 CI_95%_=[-72.8–15.9]; Fig. 2C).

Sheep: Three-four months following PVI, acellular collagenous ablation scar was observed in the RPV of sheep (Fig. 2D left panel), but not in the LPV (Fig. 2D right panel).

### 3.3 LA volume reduction in AF patients

The volume analysis was based on 20 AF patients because the CMR stack did not cover the whole atrium in 1 patient. The maximum and pre-contraction LA volume in patients decreased by 9(18) and 9(15) %, respectively, after ablation, whereas the minimum LA volume remained unchanged (Table 1). The maximum RA volume did not change after clinically successful PVI in patients (81(23) *vs.* 78(27) ml, pre- *vs.* post-ablation, CI_95%_ = [−8.2 13.2], p = 0.629).

**Table 1:** Left atrial dimensions and mechanics before and after ablation in patients.

	Pre-ablation	Post-ablation	Pre-ablation *vs.* post-ablation
Maximum LA volume, ml	103.4(37.6)	91.6(31.1)	CI_95%_ = [3.9 19.8]; p = 0.006
Pre-contraction LA volume, ml	87.9(31.3)	78.8(26.5)	CI_95%_ = [3.3 14.9]; p = 0.004
Minimum LA volume, ml	68.2[47.0]	57.9[33.6]	p = 0.391
Total LA ejection fraction, %	37.3(9.7)	32.5(10.3)	CI_95%_ = [−0.1 9.6]; p = 0.053
Active LA emptying fraction, %	26.5(10.7)	21.8(10.6)	CI_95%_ = [0.5 9.1]; p = 0.031
Passive LA conduit fraction, %	14.5(5.9)	13.7(6.4)	CI_95%_ = [−3.7 5.3]; p = 0.701
LA expansion index, %	63.0(24.7)	51.6(23.9)	CI_95%_ = [−0.1 22.9]; p = 0.051

The LA volumes were manually measured on CMR images at the moment of maximum LA dilatation, and immediately before and after atrial contraction. The mechanical parameters were calculated from these volumes. Values are expressed as mean(standard deviation) or median[interquartile range] dependent on normality. The 95% confidence intervals are included if testing was parametric.

### 3.4 Decrease in active LA emptying fraction after successful PVI in AF patients

The active LA emptying fraction decreased by 19[42] % after ablation in the patients (Table 1). The total LA ejection fraction remained stable after ablation as reflected in an unaltered passive LA conduit fraction (Table 1). We observed a tendency towards a lower LA expansion index after ablation (Table 1). Heart rate did not change in the patients (62 [13] *vs.* 66 [20] bpm; pre- *vs.* post-ablation, p = 0.170) and, therefore, did not constitute a confounding factor.

### 3.5 Decrease in PV wall contractility in AF patients

**Fig. 3:**
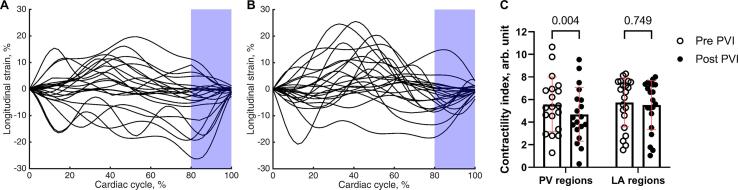
Regional wall contractility before and after ablation in patients. The longitudinal strain curves before (A) and after (B) ablation of the posterior RSPV wall where x = 0 is the moment immediately after atrial contraction. We considered the last 20% of the curve as the atrial contractile phase (blue shade). Localized contractility was quantified as the magnitude of the strain during this phase. C: The PV contractility index decreased after ablation whereas the LA contractility index remained unchanged.

Fig. 3 shows the longitudinal strain curves before (A) and after (B) ablation of the RSPV posterior wall in patients. The maximum magnitude of the last 20% of the strain curve was considered an indicative of local contractility. The PV contractility index decreased after ablation (5.57 (2.43) *vs.* 4.69 (2.38) arb. unit, pre- *vs.* post-ablation, CI_95%_ = [0.26 1.50], p = 0.004), while the LA contractility index was unaltered by PVI (5.74 (2.17) *vs.* 5.51 (2.18) arb. unit, pre- *vs.* post-ablation, CI_95%_ = [−0.37 0.82], p = 0.749; Fig. 3C).

Thus, in patients with AF, we observed that the LA volume reduction after PVI ablation was associated with a local decrease in contractility in the PV regions in which RF energy was delivered, but not in regions remote form the site of energy delivery.

## 4 Discussion

We investigated the changes in atrial dimensions and mechanics in paroxysmal AF patients after clinically successful PVI and in healthy sheep after ablation in a single PV. Our results showed that the diameter of ablated PV decreased similarly in the patients and in healthy sheep, and that a linear correlation existed between the decrease in PV dimension and extent of PV scar in patients. The maximum volume of the LA was smaller, whereas that of the RA remained unchanged, after PVI in patients. Finally, the PV walls contracted less, while the contractility of the LA walls remote from RF energy delivery did not alter after PVI in patients.

### 4.1 Reverse structural remodeling

AF causes structural remodeling of the atrial tissue by fibrosis, fat accumulation, myocardial hypertrophy, and loss of contractile fibers [1], [2], [17]. A consequence of structural remodeling is atrial dilatation that is common in AF patients and is an independent risk factor for AF in humans [3], [18]. Observations of counteracting processes in the atria have been made upon restoration of sinus rhythm, and this phenomenon has been termed reverse remodeling [13].

### 4.2 PVI-induced decrease in PV dimensions

A PVI-induced reduction in PV diameter of about 10% is observed by others [7], [10]. AF patients have enlarged PV compared with control patients, and structural remodeling due to AF burden is considered to be the cause [19]. Therefore, a reverse structural remodeling by cessation of AF has been proposed as causing the decrease in PV dimensions after successful PVI [10]. We observed that PVI ablation in healthy sheep without AF, in which structural remodeling due to arrhythmia cannot be present, caused a similar chronic decrease in PV diameter as in AF patients. It is therefore more likely that the PV size reduction is the direct causal effect of ablative energy delivery and subsequent scar formation rather than reverse remodeling processes. Indeed, we observe a statistically significant linear correlation between scar extent in the PV and PV diameter decrease after successful PVI in AF patients.

Healthy animals served as a control to the successfully ablated AF patients instead of patients with AF recurrence because in the case of AF recurrence even a persistently isolated PV (as observed on re-do catheterization) is still subject to AF remodeling (and thereby continuous dilatation). Indeed, dilatation of RSPV is observed in patients still in AF after PVI [7].

### 4.3 Ablation-induced reduction in LA volume

A meta-analysis of 8 studies supports our findings of a decrease in LA volume after successful AF ablation [8], and the LA volume decrease itself has been interpreted as a marker of reverse remodeling [9], [11], [12]. Ausma and colleagues report that 2 months of sinus rhythm (after 4 months of AF) decreased AF-induced atrial myolysis in goats, although a complete normalization was (still) not observed after 4 months of sinus rhythm [13]. This emphasizes that reverse remodeling processes does occur, but at a slow rate and most likely beyond the 3-month blinding period after AF ablation.

In patients with AF recurrence, in whom a reverse structural remodeling is not expected, a similar decrease in LA volume has been observed after AF ablation [10]. Thus, the chronic effects of ablative energy must directly have caused the LA volume reduction seen after PVI, rather than the restoration of sinus rhythm. Indeed, the volume of the RA, in which no RF energy was delivered, remained unchanged 3 months after successful PVI in our patients, although AF was equally absent as in the LA.

### 4.4 Loss of contractility at ablated sites

Our observation of a decrease in active LA emptying fraction after PVI is confirmed by others [8]. We reason that a reverse remodeling that includes normalization of the contractile fibers, should lead to an increase and not a decrease in the active LA function. An increase in active LA emptying fraction from 22 to 33% is observed after successful PVI by Marsan et al. [20]. However, 45% of these patients were medicated with angiotensin receptor blocker that is known to attenuate AF-induced atrial fibrosis [21].

We report that the contractility of 3 LA regions remote from the sites of RF delivery was unchanged after PVI. This observation questions a global reverse remodeling based on restoration of sinus rhythm. Instead, we observed a decrease in contractility in the PV walls, possibly due to loss of myocytes caused by the ablative energy. In alignment with our findings, others report a positive correlation between the amount of ablation scar (measured by late gadolinium enhancement) and a decrease in total LA ejection fraction [22].

### 4.5 Stretch-reduction as an antiarrhythmic factor

A reduction in PV and LA dimensions, under the assumption of unaltered LA pressure, decreases wall stress according to Laplace’s law. Because increased atrial and PV stretch is proarrhythmic [23], [24], and relief of chronic stretch is antiarrhythmic [25], the ablation-induced decrease in atrial dimensions may play a role in preventing AF by reduction in local wall stress. Indeed, the plasma concentration of B-type natriuretic peptide (BNP), a marker of atrial stretch, is decreased after successful PVI [11]. The authors observed a correlation between post-ablation arrhythmia burden and BNP, and they proposed BNP as a marker of reverse structural remodeling. However, it is more likely that the ablation scar decreases atrial stretch by dimension reduction and thereby correlates with a lower arrhythmia burden.

Our observation of an unaltered minimum LA volume, despite smaller maximum LA volume, after ablation indicates that PVI prevented LA dilatation by a stiffening of the chamber. Indeed, we observed a tendency towards a lower expansion index after PVI. A future goal of research is to optimize ablation therapy such that local ablative energy delivery is sufficient to decrease LA and PV dimensions to prevent proarrhythmic dilatation but small enough to prevent loss of contractile function.

### 4.6 Study limitations

The reproducibility of CMR before and after PVI prevented us from analyzing all PV. Also, the in-plane resolution was 1.25 mm × 1.25 mm in patients, and therefore the measurement of radial strain of the LA myocardial wall (changes in myocardial thickness) during the cardiac cycle was not feasible. We instead analyzed the motion fraction of the wall towards the LA center of mass.

### 4.7 Conclusion

We demonstrated that the reduction in LA and PV dimensions after clinically successful PVI is attributed to the direct effect of ablative energy delivery and scar formation rather than reverse structural remodeling based on restoration of sinus rhythm. The curative potential of reverse structural AF remodeling is therefore smaller than previously estimated. An antiarrhythmic effect of PVI may lie in the downsizing of the LA and PV.

## CRediT authorship contribution statement

**Lisa A. Gottlieb:** Conceptualization, Methodology, Software, Validation, Formal analysis, Investigation, Data curation, Writing – original draft. **Nora Al-Jefairi:** Formal analysis, Investigation, Data curation, Writing – review & editing. **Dounia El Hamrani:** Methodology, Writing – review & editing, Investigation. **Jérôme Naulin:** Methodology, Investigation. **Jérôme Lamy:** Methodology, Data curation, Software, Writing – review & editing. **Nadjia Kachenoura:** Methodology, Data curation, Software, Writing – review & editing. **Marion Constantin:** . **Bruno Quesson:** Project administration, Resources. **Hubert Cochet:** Conceptualization, Supervision, Resources. **Ruben Coronel:** Conceptualization, Writing – original draft, Funding acquisition, Validation, Supervision. **Lukas R.C. Dekker:** Conceptualization, Writing – original draft, Funding acquisition, Validation, Supervision.

## Declaration of Competing Interest

The authors declare that they have no known competing financial interests or personal relationships that could have appeared to influence the work reported in this paper.
